# Transcriptome and proteome of ovarian tissues revealed the differences in the ovaries of dairy goats during the breeding and non-breeding seasons

**DOI:** 10.3389/fvets.2025.1565807

**Published:** 2025-07-03

**Authors:** Chenbo Shi, Qingqing Liu, Wei Wang, Qiuya He, Jianqing Zhao, Fuhong Zhang, Lu Zhu, Jun Luo

**Affiliations:** Shaanxi Key Laboratory of Molecular Biology for Agriculture, College of Animal Science and Technology, Northwest A&F University, Yangling, China

**Keywords:** dairy goat, transcriptome, proteome, ovary, seasonal reproduction

## Abstract

Dairy goats represent a crucial species within global dairy livestock. In temperate regions with distinct seasons, dairy goats exhibit reduced reproductive activity under long photoperiod conditions—a phase termed the non-breeding season. This poses a significant challenge to sustaining year-round goat milk production. As the pivotal organ for reproduction, the molecular regulatory mechanisms of the ovary in seasonal breeding remain incompletely characterized. This study investigated the variations in gonadotropin levels in dairy goats across breeding and non-breeding seasons, alongside an evaluation of follicle size and quantity. Furthermore, ovarian differences were explored at the molecular level using transcriptomic and proteomic methodologies. The findings indicate that follicle-stimulating hormone (FSH) and luteinizing hormone (LH) levels in dairy goats are significantly reduced during the non-breeding season compared to the breeding season (*p* < 0.05). Furthermore, follicle sizes in dairy goats are notably larger during the breeding season relative to the non-breeding season (*p* < 0.05). A total of 1,115 differentially expressed genes (DEGs) were identified, comprising 749 upregulated and 366 downregulated genes. Additionally, 520 differentially expressed proteins (DEPs) were identified, with 162 upregulated and 358 downregulated. The identified common DEGs and DEPs exhibiting consistent expression patterns include *TMEM205*, *TM7SF2*, *SLC35G1*, *GSTM1*, and *ABHD6*. These DEGs and DEPs suppress follicular development during the non-breeding season by regulating steroid hormone biosynthesis. In conclusion, this study reveals the molecular basis underlying seasonal reproductive differences at the ovarian level in dairy goats, offering new insights into the mechanisms of their seasonal reproduction.

## Introduction

As a breed specifically developed for dairy production, the dairy goat holds a significant role within the livestock industry and the dairy sector across numerous countries (1). Goat milk is rich in essential fatty acids, abundant milk proteins, and vitamins, all of which positively influence human health (2). In regions of low latitude, such as northwestern China, dairy goats demonstrate a significant decrease in reproductive activity from spring to summer (February to August). This seasonal decline often results in fluctuations in milk supply, posing challenges in consistently meeting market and consumer demands throughout the year.

The seasonal reproductive cycle of animals is a multifaceted physiological process regulated by multiple factors, primarily including photoperiod and temperature changes, with photoperiod serving as the dominant determinant (3–6). Extended daylight periods during the non-breeding season induce inactive reproductive status in dairy goats. The photoperiod modulates melatonin secretion by the pineal gland, with light cues being relayed to the pineal gland through the suprachiasmatic nucleus in the brain. This process governs the release of thyroid stimulating hormone (TSH) from the pituitary gland via melatonin, subsequently influencing the secretion of gonadotropin-releasing hormone (GnRH) by the hypothalamus. As a result, ovarian function is affected through the actions of luteinizing hormone (LH) and follicle-stimulating hormone (FSH) secreted by the pituitary gland (7). In certain mammalian species, alterations in photoperiod can modulate the activity of kisspeptin and RFRP-3 neurons within the hypothalamus, which are integral to the regulation of GnRH release. Consequently, photoperiodic changes influence the functionality of these neurons, thereby impacting the reproductive axis and dictating the timing of seasonal reproduction (8).

Estrogen (E2) and progesterone (P4) secreted by the ovaries play a central role in regulating the reproductive cycle and supporting pregnancy, while the ability of the ovaries to store oocytes directly affects female fertility (9). Notable differences exist in ovarian biological function between breeding and non-breeding seasons. Research indicates that reduced LH levels during the non-breeding season can result in slower follicular development (10). In Boer goats, the size and number of follicles during the non-breeding season are diminished compared to the breeding season (11). However, the alterations in ovarian function during the non-breeding season are intricate and necessitate further investigation into their underlying causes.

RNA sequencing (RNA-seq) provides valuable insights into genetic information at the transcriptional level. Furthermore, proteomic analysis of functional proteins enables more comprehensive elucidation of underlying molecular mechanisms. In this study, we investigated follicular development and reproductive hormone levels in dairy goats during both the breeding and non-breeding seasons to enhance our understanding of their reproductive status. Additionally, RNA-seq was employed to compare the ovarian transcriptomic profiles of dairy goats, while a 4D-DIA proteomic approach was utilized to examine the ovarian proteomic data. This dual analysis offers insights into the transcriptomic and proteomic variations in ovarian expression patterns across different seasons. The findings provide a foundation for elucidating the specific characteristics of seasonal reproduction in dairy goats and for exploring potential underlying molecular mechanisms.

## Materials and methods

The experiment was performed under approval by the Animal Ethical and Welfare Committee at the Northwest Agriculture and Forestry University, China.

### Animals

This study was conducted in region, Shaanxi, China (34° 16′N, 108° 4′E). The region has distinct four seasons, characterized by a continental monsoon climate, with an average annual temperature of 12°C. Following the summer solstice, the duration of daylight diminishes, prompting the dairy goats in the region to enter the breeding season. Conversely, post the winter solstice, the daylight duration increases, leading the dairy goats in the area to transition into the non-breeding season. The peak breeding season for dairy goats in the region is from August (14 h of daylight) to October (12 h of daylight), while clearly exhibit anestrus status from March (12 h of daylight) to May (14 h of daylight).

The goat breed is Xinnong Saanen dairy goats, all females were multiparous and ranged in age from 2.5 to 3.5 years, with an average body weight of 47.29 ± 7.45 kg. The diet consists of corn silage and alfalfa hay for roughage. The concentrate includes soybean meal, corn grain, bran, trace mineral salt, and vitamin-mineral premix. The concentrate-to-roughage ratio is 4:6, and they have ad libitum access to water.

### Experimental design

Forty dairy goats were divided into two experimental groups, with studies conducted during the non-breeding season group (NBS group) from March to May and the breeding season group (BS group) from August to October, each lasting 6 weeks. Weekly blood samples were obtained from the goats via jugular venipuncture. Upon the conclusion of the experiment, ovarian samples from the goats were procured for subsequent analysis.

### Ovaries collection and follicle counting

The ovaries were harvested under general anesthesia with xylazine hydrochloride (Administer a muscular injection at a dose of 2.5 μL/kg in accordance with the instructions; Huamu Animal Health Products Corporation), and the ovaries were collected in a sterile operating room. Following the surgery, the goats were placed in quiet and clean pens for 7 days, with daily iodine disinfection of the wounds. The follicle count was recorded, and the diameter of each follicle was measured using a ruler. Follicles with a diameter smaller than 2.5 mm and larger than 2.5 mm were categorized as small-sized and large-sized follicles, respectively. The ovarian tissues were preserved in liquid nitrogen for subsequent transcriptomic and proteomic sequencing analyses.

### RNA sequencing

The total RNA of the ovaries was extracted by Trizol reagent (Invitrogen, United States), according to the instructions of the manufacturer. The purity and concentration of the extracted RNA were detected by NanoDrop 2000A Spectrophotometer (Thermo Fisher Scientific, United States). According to the manufacturer’s manual, cDNA libraries and sequencing were performed at Allwegene Company (Beijing, China). Briefly, mRNAs were extracted from total RNA using oligo (dT) magnetic beads and then broken into short fragments. The fragmented mRNAs were reverse-transcribed into cDNAs which were then amplified by PCR.

The cDNA libraries were sequenced using Illumina HiSeq 2000. Clean data were obtained by removing reads containing adapters and low-quality reads from the raw data. Subsequent analyses were performed using the clean data. The clean redas were mapped to the *Capra hircus* reference genome Capra_hircus. ARS1.^1^ Calculate the number of clean reads mapped to each gene, and standardize gene expression using the RPKM (Reads per kilobase per million mapped reads) method. Based on RPKM-normalized results, gene expression levels are evaluated, with genes satisfying *p* < 0.05 and |FoldChange| > 2identified as differentially expressed genes (DEGs). CPM (Counts per million mapped reads) data are used to assess the quality and accuracy of transcriptomic analyses.

R software (Ggplot2 R package) was used for plot, and Kyoto Encyclopedia of Genes and Genomes (KEGG) enrichment analysis was performed using the OmicShare tool.^2^ Gene Ontology (GO) enrichment analysis was performed and the results visualized with the OmicShare tool, an online platform for data analysis.^3^ KEGG pathways or GO terms with *p* < 0.05 were considered significantly enriched in DEGs.

### 4D-DIA proteomics

Ovarian samples were retrieved from liquid nitrogen storage and pulverized using liquid nitrogen. Subsequently, 200 mg of the pulverized sample was transferred to a centrifuge tube, and 300 μL of RIPA buffer solution was added and mixed thoroughly. Cryogenic grinding was performed using steel beads at 70 Hz for 4 min, followed by centrifugation at 12,000 rpm for 10 min at 4°C. The samples then underwent ultrasonic treatment in an ice-water bath for 15 min to ensure complete lysis. A second centrifugation was conducted at 12,000 rpm for 10 min at 4°C, and the supernatant was collected for further analysis.

The protein concentration was quantified utilizing the Bicinchoninic Acid (BCA) assay. Subsequently, peptide samples were prepared for instrumental analysis. In summary, proteins underwent precipitation via acetone, followed by reconstitution, reduction, and alkylation. Trypsin was then employed for enzymatic digestion of the proteins. Subsequently, trifluoroacetic acid (TFA) was used to remove sodium deoxycholate (SDC) from the peptide samples. Following desalting, the samples were prepared for instrumental analysis.

For each sample, 200 ng of total peptides were separated and analyzed with a nano-UPLC (nanoElute2) coupled to a timsTOF Pro2 instrument (Bruker) with a nano-electrospray ion source. Vendor’s raw MS files were processed using SpectroMine software (4.2.230428.52329) and the built-in Pulsar search engine.

The statistical analysis of the dataset was primarily executed utilizing R software (version 4.0). Protein raw intensity values underwent normalization via the median. The identification of significantly different proteins was conducted using the R package metaX, applying a filtration based on two criteria: a *p*-value < 0.05 from the T-test and a fold change > 1.2. Enrichment analyses for GO and KEGG Pathways of the differentially expressed proteins were performed using hypergeometric tests, with functional terms deemed significantly enriched if they exhibited a *p*-value < 0.05. The subcellular localization of proteins was analyzed using the WoLF PSORT software.^4^

### Real-time quantitative PCR

Extract total RNA using the previously described method. Subsequently, 1,000 ng of RNA was reverse transcribed into cDNA utilizing the PrimeScript™ RT reagent Kit with gDNA Eraser (Takara, China). The sequences and GenBank accession numbers of the primers employed for target gene amplification are presented in Table 1. Quantitative detection of the target genes was performed using the SYBR Green PCR Master Mix (Takara, China). The relative gene expression was analyzed using the 2 − ΔΔCt method.

**Table 1:** Primers used for the qRT-PCR validation.

Gene name	Primer sequence (5′-3′)	Product size (bp)	Accession number
FASN	F: GCACACAATATGGACCCCCA	183	NM_001285629.1
	R: CATGCTGTAGCCTACGAGGG		
LDLR	F: TTTGAACGCACGAAAGCAGG	165	XM_005682375.3
	R: GTTTGATCCTGACCAGGGGG		
FSHR	F: ATGCGGTCGAACTGAGGTTT	146	NM_001285636.1
	R: GGGCAGGTTGGAGAACACAT		
TMEM206	F: ACTGAGCAGCCACCTACCTA	116	XM_018060751.1
	R: TGAGGAGGGACCTACTCTGC		
TM7SF2	F: CCTACCGAATCGTGCCCTAC	176	XM_005699882.3
	R: CCTATCAGGCCGTTCATCCC		
SLC35G1	F: CTGTCCTGGACTTGGCTTGT	192	XM_018041634.1
	R: CGTTGACCTTTGGGGCCTAT		
ABHD6	F: GAGGTCAGCCCCTCTAGGTA	175	XM_018038385.1
	R: TCCTCCGCCAGTACCAGTAA		

### Elisa assay

Centrifuge the blood samples at 3000 rpm for 5 min to collect the serum. Use the ELISA kit (Fankew, China) according to the manufacturer’s instructions to detect FSH and LH in the serum.

### Statistical analyses

Statistical analyses were conducted utilizing SPSS software version 17.0 (SPSS Inc., Chicago, IL, United States), incorporating a minimum of three independent replicates. Independent sample t-tests were employed to compare the two groups, with all data expressed as mean ± standard error of the mean (S. E. M.). A *p*-value of less than 0.05 was deemed statistically significant. The levels of significance are denoted as follows: * indicates *p* < 0.05, ** indicates *p* < 0.01, and *** indicates *p* < 0.001.

## Results

### Reproductive hormone levels and follicle counts

**Figure 1:**
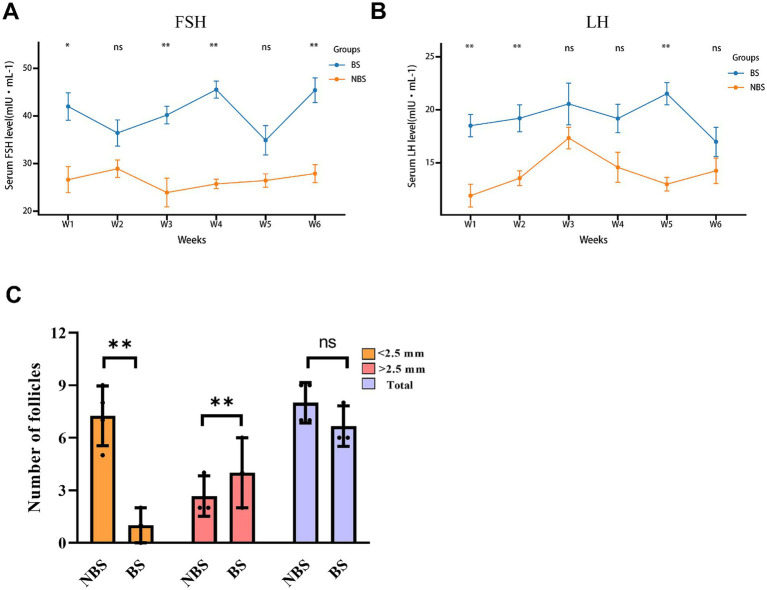
Levels of FSH and LH in the serum and follicle count in dairy goats during the breeding and non-breeding seasons. The contents of FSH **(A)** and LH **(B)** in serum were measured by ELISA. **(C)** Size and number of ovarian follicles.*p < 0.05, **p < 0.01, ns > 0.05.

During the six-week experimental period, the levels of FSH and LH in dairy goats were assessed weekly. Serum FSH levels during weeks 1, 3, 4, and 6 of the breeding season were significantly higher than those in the non-breeding season (*p* < 0.05), while serum LH levels during weeks 1, 2, and 5 of the breeding season were significantly higher than those in the non-breeding season (*p* < 0.05; Figures 1A,B). Despite these findings, FSH levels at weeks 2 and 5 showed no significant difference (*p* > 0.05), and LH levels at weeks 3, 4, and 6 also exhibited no significant difference (*p* > 0.05; Figures 1A,B). Additionally, an analysis of follicular size and quantity indicated that, relative to the non-breeding season, dairy goats in the breeding season exhibited a greater number of large-sized follicles (*p* < 0.05) and fewer small-sized follicles (*p* < 0.05), while the total follicle count remained statistically unchanged (*p* > 0.05; Figure 1C).

### Transcriptome analysis

**Figure 2:**
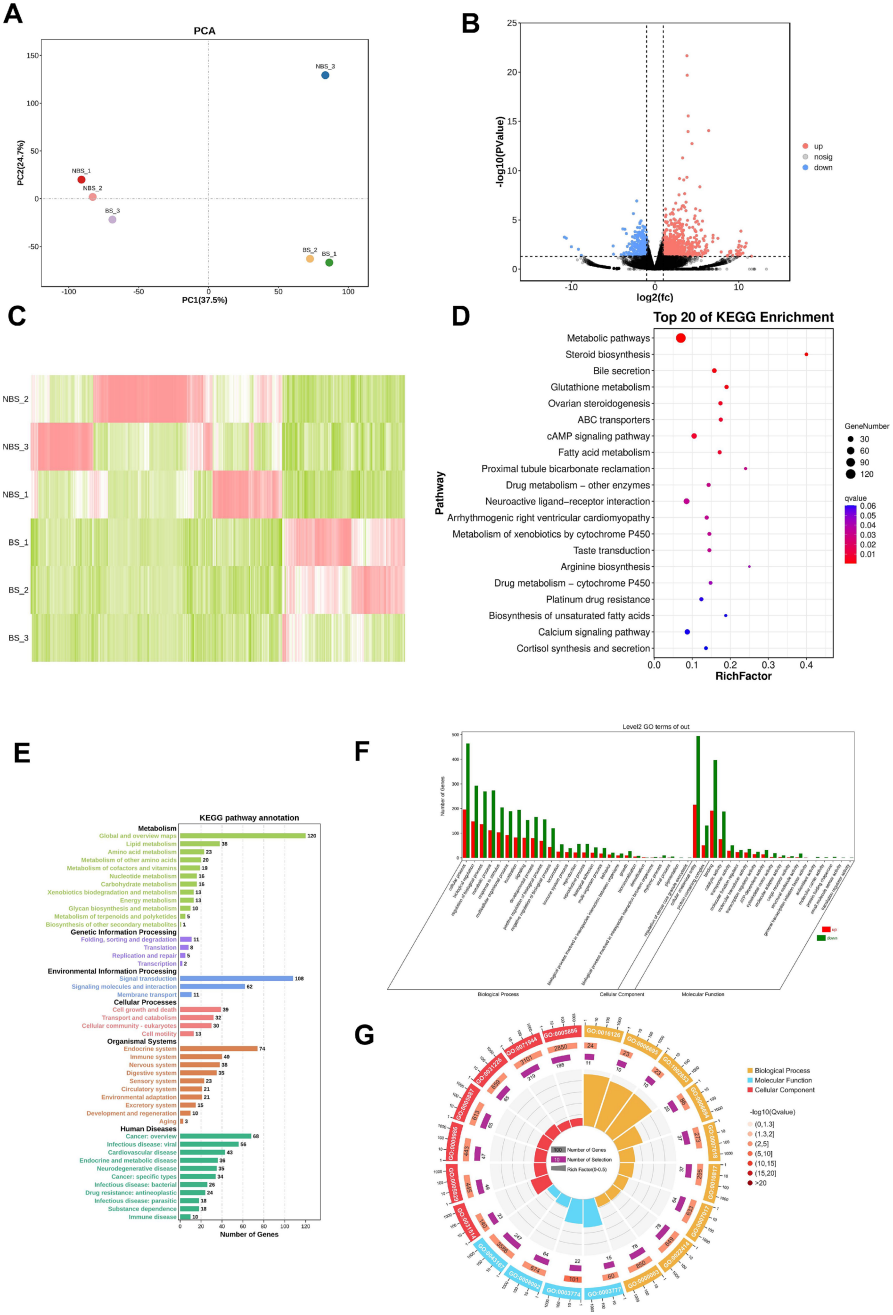
Transcriptome sequencing and bioinformatics analysis. **(A)** PCA analysis. **(B)** Volcano plot. **(C)** Heatmap of DEGs. **(D)** Top 20 pathways obtained from KEGG pathway enrichment. **(E)** KEGG pathway annotation. **(F)** GO terms enrichment analysis. **(G)** Enrichment circle plot of GO terms.

The principal component analysis (PCA) results illustrate the distribution of samples across the first two principal components (PC1 and PC2), with PC1 accounting for 37.5% of the variance and PC2 accounting for 24.7% (Figure 2A). A total of 954 DEGs were identified between the NBS and BS groups, with 749 genes upregulated in the NBS group and 366 genes upregulated in the BS group (Figures 2B,C). A comprehensive analysis of the top 20 DEGs revealed that the log2(fc) values for integrin subunit beta 6 (ITGB6), fibrous sheath interacting protein 2 (FSIP2), and acid phosphatase 3 (ACP3) were upregulated by factors of 10.9, 10.7, and 10.5, respectively (Table 2). Conversely, the log2(fc) values for G protein-coupled Receptor 22 (GPR22) and pyroglutamylated RFamide peptide receptor (QRFPR) were downregulated by factors of 10.6 and 10.0, respectively (Table 2).

**Table 2:** The top 20 upregulated or downregulated DEGs in NBS vs. BS.

Ensemble ID	Symbol	Description	Log2(fc)	*p*-value	UP/DOWN
ENSCHIG00000004135	–	–	11.5	0.048	UP
ENSCHIG00000013972	ITGB6	Integrin beta-6	10.9	0.002	UP
ENSCHIG00000020565	-	-	−10.8	0.001	DOWM
ENSCHIG00000016918	-	-	10.8	0.005	UP
ENSCHIG00000016003	FSIP2	Fibrous sheath interacting protein 2	10.7	0.006	UP
ENSCHIG00000010247	GPR22	G protein-coupled receptor 22	−10.6	0.001	DOWM
ENSCHIG00000016695	ACP3	Acid phosphatase 3	10.5	0.023	UP
ENSCHIG00000010784	RNASE12	Ribonuclease A family member 12	10.5	0.024	UP
ENSCHIG00000026746	-	-	10.5	0.024	UP
ENSCHIG00000021440	CNNM1	Cyclin-M1	10.3	0.005	UP
ENSCHIG00000016148	ANO2	Anoctamin 2	10.3	0.000	UP
ENSCHIG00000022191	ERN2	Endoplasmic reticulum nucleotide receptor 2	10.2	0.011	UP
ENSCHIG00000004467	-	-	10.2	0.035	UP
ENSCHIG00000002414	ACTL7A	Actin like 7A	10.2	0.025	UP
ENSCHIG00000015989	-	-	10.1	0.001	UP
ENSCHIG00000007733	GJB1	Gap junction protein beta 1	10.0	0.004	UP
ENSCHIG00000015444	SCGN	Secretagogin	10.0	0.045	UP
ENSCHIG00000022770	P2RX2	Purinergic receptor P2X 2	10.0	0.020	UP
ENSCHIG00000026596	QRFPR	Pyroglutamylated RFamide peptide receptor	−10.0	0.005	DOWM
ENSCHIG00000008489	FGFBP1	Fibroblast growth factor binding protein 1	10.0	0.006	UP

KEGG enrichment analysis identified the top 20 pathways significantly enriched with DEGs (Figure 2D). Among these, 120 DEGs were significantly enriched in metabolic pathways, 8 DEGs in steroid biosynthesis, 8 DEGs in glutathione metabolism, and 12 DEGs in ovarian steroidogenesis. The enriched pathways are predominantly annotated in categories such as metabolism, genetic information processing, environmental information processing, cellular processes, organizational systems, and human diseases (Figure 2E). Notably, metabolism accounts for the highest number of annotated pathways (12 pathways), whereas environmental information processing accounts for the fewest (only 3 pathways; Figure 2E).

GO analysis identified the top 20 terms that are significantly enriched among the DEGs, categorizing them into three primary domains: biological process, molecular function, and cellular component (Figures 2F,G). The terms exhibiting the highest distribution of DEGs include cellular anatomical entity, cellular process, binding, and biological regulation (Figures 2F,G).

### Proteomic analysis

**Figure 3:**
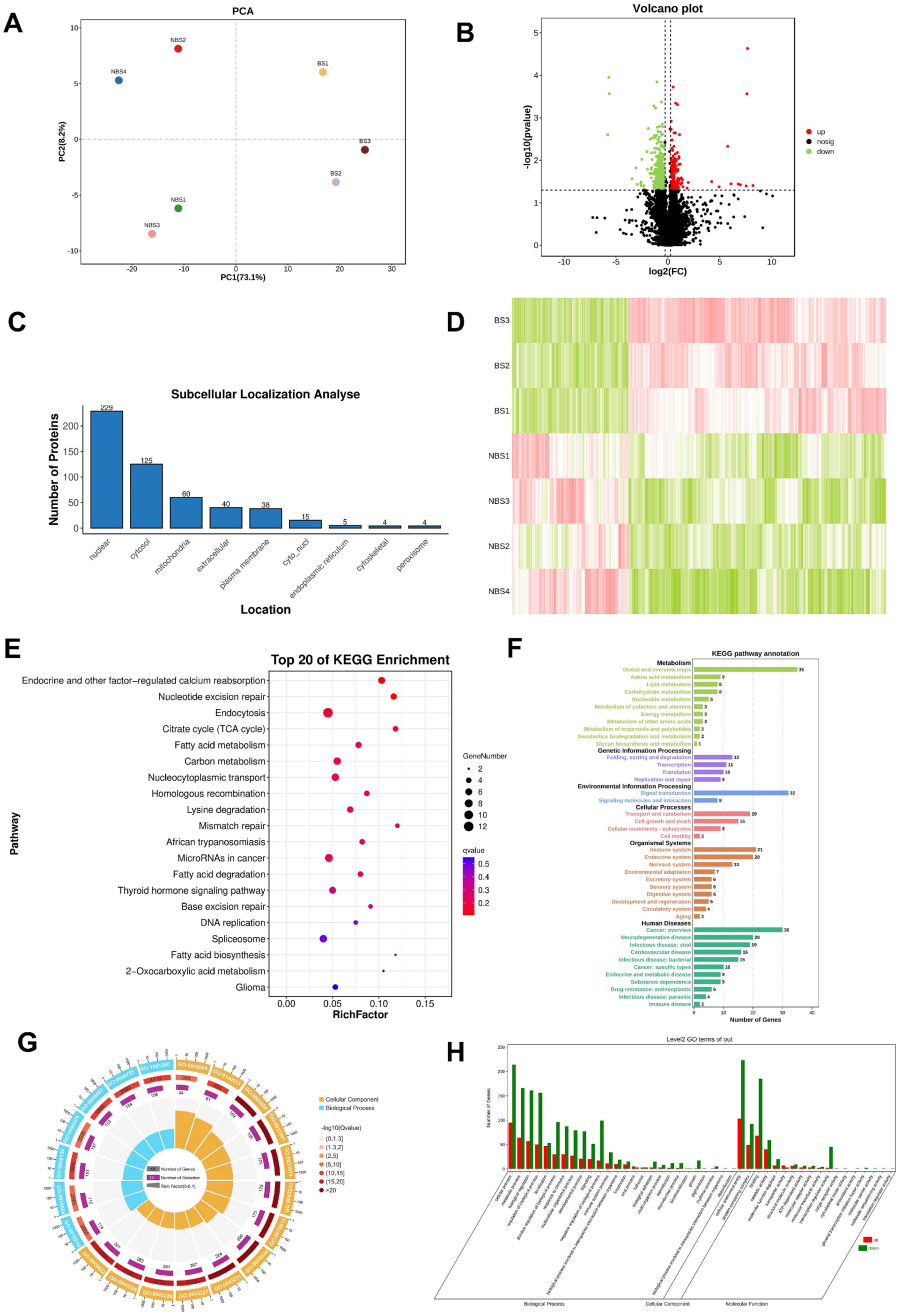
Proteomic sequencing and bioinformatics analysis. **(A)** PCA analysis. **(B)** Volcano plot. **(C)** Subcellular localization of DEPs. **(D)** Heatmap of DEPs. **(E)** Top 20 pathways obtained from KEGG pathway enrichment. **(F)** KEGG pathway annotation. **(G)** Enrichment circle plot of GO terms. **(H)** GO terms enrichment analysis.

The results of 4D-DIA proteomics analysis showed that principal component analysis (PCA) revealed the distribution of principal components, with PC1 accounting for 73.1% of the variance and PC2 for 8.2% (Figure 3A). In comparison to NBS group, the BS group exhibited 162 upregulated and 358 downregulated differential expressed proteins(DEPs), as illustrated in the volcano plot and heatmap (Figures 3B,D). Subcellular localization analysis revealed that the DEPs are predominantly situated in the nucleus, cytoplasm, and mitochondria (Figure 3C). Table 3 presents the log2 fold change (fc) values for the top 20 DEPs, highlighting that LOC108633460, solute carrier family 35 member (SLC35G1), and grancalcin (GCA) were upregulated by 8.20, 7.67, and 7.71 times, respectively. Conversely, the log2 (fc) values for LysM domain containing 3 (LYSMD3) and histone acetyltransferase (KAT2A) were reduced by 5.69 and 5.64 times, respectively (Table 3).

**Table 3:** The top 20 upregulated or downregulated DEPs in NBS vs. BS.

UniProt	Gene name	Description	Log2(fc)	P-Value	UP/DOWN
A0A452E5B3	LOC108633460	-	8.20	0.039	UP
A0A452EUQ3	SLC35G1	Solute carrier family 35 member G1	7.67	<0.0001	UP
A0A452G0K2	GCA	Grancalcin	7.61	<0.0001	UP
A0A452FHU2	-	-	7.55	0.040	UP
A0A452ET71	MPC1	Mitochondrial pyruvate carrier	6.98	0.037	UP
A0A452FW07	AMDHD1	Amidohydrolase domain-containing protein 1	6.78	0.036	UP
A0A097ITJ2	DSG4	Desmoglein 4	6.08	0.036	UP
A0A8C2S393	-	-	−5.79	0.003	DOWN
A0A452EFZ7	-	-	5.77	0.005	UP
A0A452DPQ3	LYSMD3	LysM domain containing 3	−5.69	<0.0001	DOWN
A0A452FHR7	KAT2A	Lysine acetyltransferase 2A	−5.64	0.000	DOWN
A0A452F5C7	LPIN1	Phosphatidate phosphatase	4.93	0.042	UP
A0A452F944	ABLIM2	Actin binding LIM protein family member 2	4.22	0.032	UP
A0AAJ7P8Z1	MAP4	Microtubule-associated protein	−3.45	0.027	DOWN
A0A452G561	YAF2	YY1 associated factor 2	−3.07	0.015	DOWN
A0A452FBP5	VGLL4	Vestigial like family member 4	−2.96	0.037	DOWN
A0A452FRZ1	LOC108638216	-	−2.62	0.035	DOWN
A0A8C2S6S3	RAB34	RAB34, member RAS oncogene family	−2.58	0.032	DOWN
A0A452G1X2	UBE2S	E2 ubiquitin-conjugating enzyme	−2.58	0.039	DOWN
A0A452FDM1	-	-	−2.51	0.009	DOWN

KEGG enrichment analysis identifies the top 20 enriched pathways, with the five most significant being endocrine and other factor-regulated calcium reabsorption, nucleotide excision repair, endocytosis, citrate cycle (TCA Cycle), and fatty acid metabolism. Additionally, the thyroid hormone signaling pathway, associated with steroid hormone synthesis, is also enriched (Figure 3E). Pathway annotation results indicate that there are 11 pathways each annotated to metabolism and human diseases (Figure 3F).

Furthermore, Gene Ontology (GO) analysis identifies the top 20 significantly enriched terms among the DEPs, with cellular anatomical entity, cellular process, and binding exhibiting the highest distribution of DEPs. These top 20 terms are categorized exclusively under cellular component and biological process, with no classification under molecular function (Figures 3G,H).

### Conjoint analysis of DEGs and DEPs

**Figure 4:**
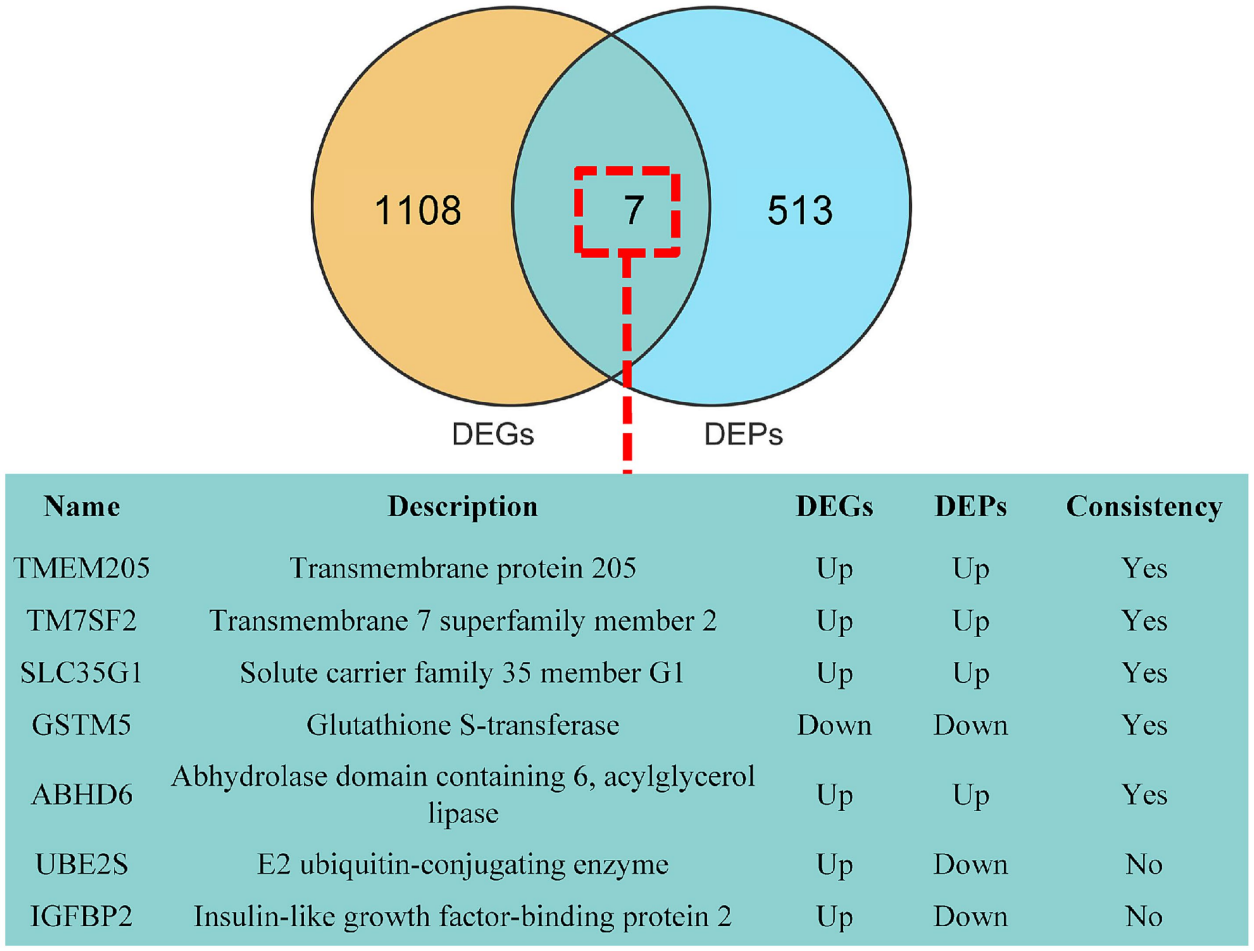
Conjoint Analysis of DEGs and DEPs. There are 7 shared DEGs/DEPs (green).

To overcome the constraints of analyzing a single omics dataset and to improve the precision and dependability of bioinformatics analysis, a joint analysis of DEGs and DEPs was conducted. Seven common DEGs/DEPs were identified, with transmembrane protein 205 (TMEM205), transmembrane 7 superfamily member 2 (TM7SF2), solute carrier family 35 member G1 (SLC35G1), glutathione S-transferase (GSTM5), abhydrolase domain containing 6, acylglycerol lipase (ABHD6), E2 ubiquitin-conjugating enzyme (UBE2S) and insulin-like growth factor-binding protein 2 (IGFBP2)exhibiting similar expression patterns in both DEGs and DEPs (Figure 4).

### Validation of selected DEGs by qRT-PCR

**Figure 5:**
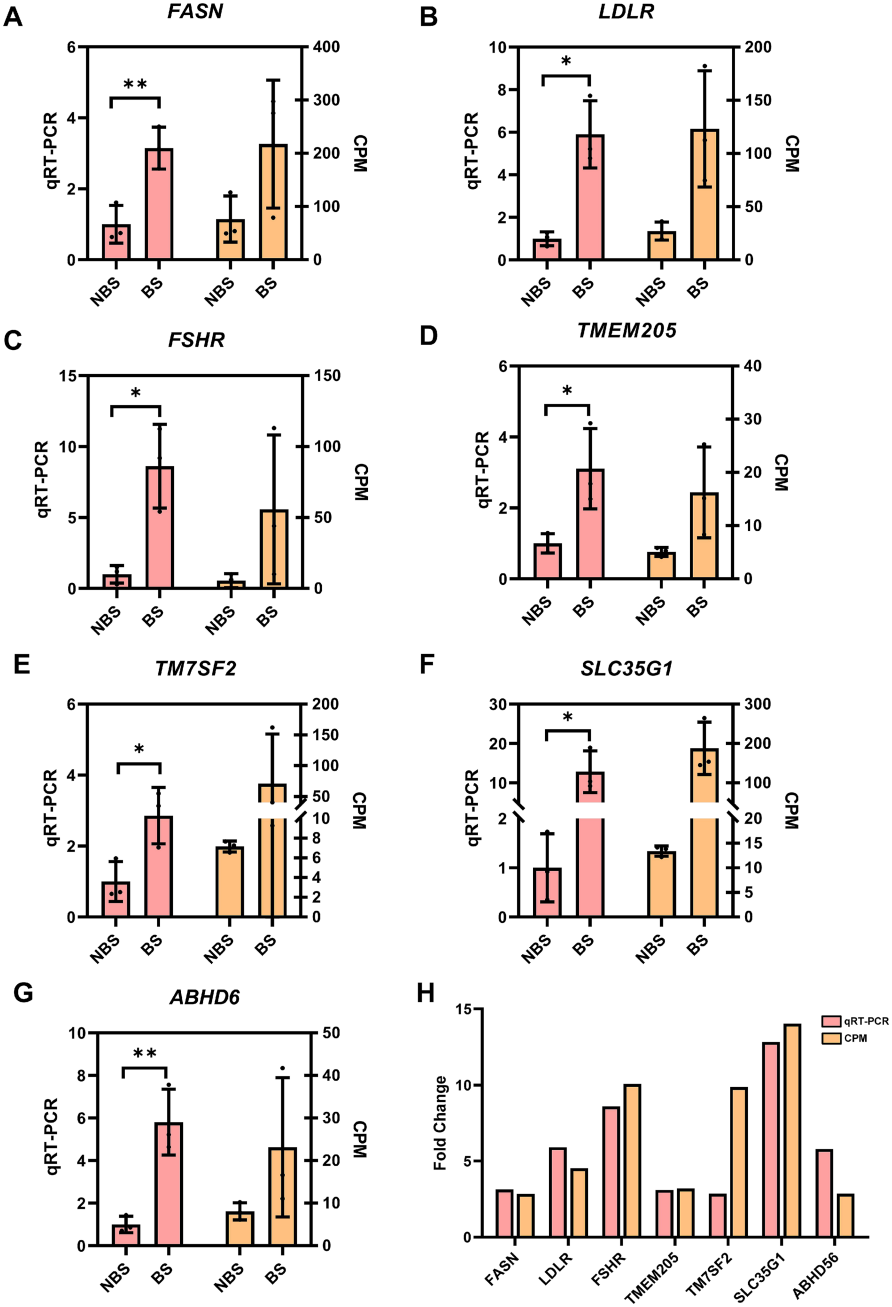
The expression of some DEGs were verified by qRT-PCR. The CPM value(right Y-axis) was taken as the relative expression level of DEGs(left Y-axiss). **(A)** *FASN*. **(B)** *LDLR*. **(C)** *FSHR*. **(D)** *TMEM205*. **(E)** *TM7SF2*. **(F)** *SLC35G1*. **(G)** *ABHD6*. **(H)** Fold change between qRT-PCR and CPM. The relative expression level of DEGs were analyzed with independent sample t-tests.*p < 0.05, **p < 0.01.

qRT-PCR validation was conducted on genes associated with ovarian hormone synthesis, in addition to the four shared genes identified from DEGs and DEPs. In the ovarian tissues of dairy goats during the breeding season, the expression levels of fatty acid synthase (FASN) (*p* < 0.01), low-density lipoprotein receptor (LDLR) (*p* < 0.05), follicle stimulating hormone receptor (FSHR) (*p* < 0.05), TMEM205 (*p* < 0.05), TM7SF2 (*p* < 0.05), ABHD6 (*p* < 0.01) and SLC35G1 (*p* < 0.05) were significantly increased (Figures 5A–G), and the fold change showed a trend similar to the corresponding counts per million (CPM) values in the RNA sequencing results, indicating the accuracy of the RNA sequencing data (Figure 5H).

## Discussion

The regulation of reproductive hormones, including FSH and LH, is a multifaceted process influenced by a range of factors, encompassing environmental cues and intrinsic physiological mechanisms. In seasonally breeding species such as goats, the secretion of these hormones is typically downregulated during the non-breeding season, resulting in diminished reproductive activity. The seasonality of goat reproduction is primarily governed by alterations in photoperiod, which modulate the negative feedback exerted by estradiol on LH secretion. Specifically, during extended photoperiods, the negative feedback of E2 is enhanced, leading to reduced LH secretion and the suppression of ovulation (12, 13). Similarly, during the non-breeding season, goats exhibit decreased levels of FSH (14). The administration of PMSG or other pharmacological agents that stimulate FSH release can induce ovulation in goats during this period. In this study, we observed that the overall concentrations of FSH and LH in the bloodstream of dairy goats during the non-breeding season are significantly lower than those observed during the breeding season. FSH and LH are pivotal gonadotropins that regulate various dimensions of ovarian follicular development, including follicular growth, maturation, and ovulation. FSH is particularly critical for follicle maturation and development, whereas LH plays a significant role in follicular development and oocyte maturation (15, 16). Furthermore, existing research suggests that follicular development is subject to seasonal influences (17, 18). Our study reveals that follicle size in dairy goats is reduced during the non-breeding season compared to the breeding season, which we attribute to diminished levels of FSH and LH during the non-breeding period. Consequently, the analysis of hormone concentrations and follicle counts indicates that the ovarian activity of dairy goats is in a quiescent state during the non-breeding season relative to the breeding season.

To further investigate the potential genetic factors underlying the differences in seasonal reproductive status, we conducted transcriptomic analysis and 4D-DIA proteomic analysis, identifying several DEGs and DEPs that may be related to ovarian function. In the study of ovarian aging, *ITGB6* is associated with the remodeling of the extracellular matrix, which may affect the fibrotic process of the ovary. Studies have shown that *ITGB6* is highly expressed in the stromal cells of aged ovaries and is associated with the cell-stromal junction process (19). In addition, previous studies have indicated that ITGB6 promotes lipid metabolism in ovarian cancer through its interaction with the PI3K signaling pathway (20). GPR22 is classified as a G protein-coupled receptor. Although research on the role of GPR22 in ovarian function is still limited, existing evidence indicates the involvement of G protein-coupled receptors in follicular development, ovulation, and ovarian steroid hormone metabolism. Within the ovarian context, the activation of the G protein-coupled estrogen receptor (GPER) is intimately associated with critical processes such as cell proliferation, differentiation, and apoptosis, all of which are essential for oocyte growth and ovulation (21). Additionally, G protein-coupled receptor 30 (GPR30) has been identified as a regulatory factor in the secretion of gonadotropins in cattle, underscoring its potential importance in reproductive regulation (22). *SLC35G1* was significantly upregulated in granulosa cells of different sizes of follicles compared to follicular membrane cells (23). It was found that the leukemia inhibitory factor promoted the expression of *KAT2A in vitro* maturation of bovine oocytes (24). The KEGG enrichment analysis reveals that the DEGs and DEPs are predominantly associated with metabolic pathways and pathways related to steroid synthesis. Metabolic pathways are integral to ovarian hormone secretion and follicular development, with metabolic status exerting a significant influence on ovarian function. This is particularly pertinent as metabolic pathways are intrinsically linked to follicle maturation (25). During follicular development, the synthesis of steroid hormones, such as estradiol and progesterone, is facilitated by both granulosa and theca cells, with secretion levels being directly modulated by FSH and LH (26). In the initial stages of follicular development, FSH enhances the expression of the steroidogenic acute regulatory protein (StAR) in granulosa cells via its receptors, thereby augmenting the transport of cholesterol to the mitochondria—a critical step in steroid synthesis (27). Concurrently, LH, through its receptors in theca cells, upregulates the expression of steroidogenic enzymes, thereby promoting the synthesis of steroid hormones (26). Our GO analysis reveals a significant enrichment of terms related to cellular processes and biological regulation among the differentially expressed genes, with a particular emphasis on those associated with ovarian development and hormone secretion. In the context of follicular development, ovarian granulosa cells engage in bidirectional communication with oocytes, a process that is crucial for the development and functionality of both cell types. Current research suggests that the interactiono between granulosa cells and oocytes can elucidate changes in gene expression during follicle maturation, as evidenced by transcriptomic analyses (28).

The integration of multi-omics analyses offers insights into various tiers of biological systems. Our comprehensive analysis of DEGs and DEPs revealed 5 common DEGs/DEPs, which demonstrated consistent expression patterns in both the transcriptomic data and RT-qPCR validation. This concordance underscores the reliability of the transcriptomic findings. The *TMEM205* gene, which encodes the transmembrane protein 205, is a member of the transmembrane protein family (29). Although research on this gene is currently limited, evidence suggests that mutations in *TMEM205* may be associated with chemotherapy resistance in ovarian cancer (30). Research has shown that *TM7SF2* is involved in the steroidogenic biosynthetic pathways of ovarian granulosa cells (31). Combining this with our results, it can be inferred that TM7SF2 may influence follicle maturation by affecting hormone synthesis in granulosa cells. Additionally, *SLC35G1* is expressed in granulosa cells, with expression levels in follicles of varying sizes surpassing those in theca cells (23). This suggests that SLC35G1 may contribute to the functional differences in synthesis between granulosa and theca cells, functions that are tightly regulated by FSH and LH, potentially due to seasonal variations in LH and FSH levels in dairy goats. Research has demonstrated that human subjects possessing the *GSTM1* deletion genotype exhibit significantly reduced testosterone levels (32). This phenomenon may be attributed to the role of GSTM1 as a steroid-binding protein, implying that GSTM1 potentially regulates steroid synthesis and thereby influences the seasonal reproductive status of dairy goats. Conversely, limited research exists regarding the role of ABHD6 in ovarian function. Research on *ABHD6* and ovarian function is scarce; however, some studies have confirmed that estrogen can promote high expression of *ABHD6* in women immune cells (33). There is a wealth of research indicating that estrogen can influence estrus in goats (34, 35). This suggests that the secretion of ovarian estrogen may differ between the breeding and non-breeding seasons.

## Conclusion

In conclusion, this study utilized transcriptomic and proteomic sequencing to investigate the molecular basis of seasonal reproductive differences in dairy goats. We, respectively, identified 1,115 DEGs and 520 DEPs in ovarian tissue between the breeding and non-breeding seasons. Critically, our results demonstrate reduced FSH and LH levels and impaired follicular development during the non-breeding season. Furthermore, functional analyses strongly suggest that the genes *TMEM205*, *TM7SF2*, *SLC35G1*, *GSTM1*, and *ABHD6* contribute to suppressed ovarian function in the non-breeding season, through regulating specific roles in steroid hormone synthesis pathways. These findings offer new insights into the molecular mechanisms underlying seasonal variations in dairy goat ovaries, presenting innovative strategies to mitigate seasonal anestrus and enhance year-round milk production.

## Data Availability

The transcriptomics datasets generated during the current study are available in NCBI SRA (PRJNA1280907), https://www.ncbi.nlm.nih.gov/bioproject/PRJNA1280907. The mass spectrometry proteomics data have been deposited to the ProteomeXchange Consortium, https://proteomecentral.proteomexchange.org, via the iProX partner repository with the dataset identifier PXD065416.

## Footnotes

^1^ https://ftp.ensembl.org/pub/release-111/fasta/capra_hircus

^2^ https://www.omicshare.com/tools/home/report/koenrich.html

^3^ https://www.omicshare.com/tools/home/soft/enrich_circle

^4^ https://wolfpsort.hgc.jp/
